# Concordance between microsatellite instability and mismatch repair protein expression in colorectal cancer and their clinicopathological characteristics: a retrospective analysis of 502 cases

**DOI:** 10.3389/fonc.2023.1178772

**Published:** 2023-06-22

**Authors:** Meihua Ye, Guoqing Ru, Hang Yuan, Lili Qian, Xianglei He, Shuangshuang Li

**Affiliations:** ^1^ Cancer Center, Department of Pathology, Zhejiang Provincial People’s Hospital, Affiliated People’s Hospital, Hangzhou Medical College, Hangzhou, Zhejiang, China; ^2^ General Surgery, Cancer Center, Department of Colorectal Surgery, Zhejiang Provincial People’s Hospital Affiliated People’s Hospital, Hangzhou Medical College, Hangzhou, Zhejiang, China; ^3^ Key Laboratory of Tumor Molecular Diagnosis and Individualized Medicine of Zhejiang Province, Hangzhou, Zhejiang, China; ^4^ Cancer Center, Department of Hematology, Zhejiang Provincial People’s Hospital, Affiliated People’s Hospital, Hangzhou Medical College, Hangzhou, Zhejiang, China

**Keywords:** MSI, MMR, CRC, concordance, clinicopathological characteristics, testing echelon

## Abstract

Microsatellite instability (MSI) is one of the hallmarks of colorectal cancer (CRC). Mismatch repair (MMR) protein expression may reflect MSI status. To analyze the concordance between MSI and MMR expression in CRC and their clinicopathological characteristics, 502 CRC patients were retrospectively collected in this study. Polymerase chain reaction-capillary electrophoresis (PCR-CE) was used to measure MSI, and MMR expression was determined by immunohistochemistry (IHC). The causes of non-concordance were analyzed. Chi-square test was used to find the correlation between MSI and various clinicopathological parameters. PCR-CE results showed 64 (12.7%) patients had high microsatellite instability (MSI-H); low microsatellite instability (MSI-L) and microsatellite stable (MSS) cases were 19 (3.8%)and 419 (83.5%), respectively. With regard to IHC, 430 (85.7%) showed proficient mismatch repair (pMMR) and 72 (14.3%) showed deficient mismatch repair (dMMR). The coincidence rate of MSI and MMR expression in CRC was 98.4% (494/502), with good concordance (Kappa = 0.932). Using PCR-CE as the gold standard, the sensitivity, specificity, positive predictive value, and negative predictive value of IHC were 100%, 98.2%, 88.9%, and 100%, respectively. MSI-H was more common in women, right colon, tumors ≥ 5 cm, ulcerative type, mucinous adenocarcinoma, poor differentiation, T stage I/II, and without lymph node or distant metastasis for CRC patients. In summary, MSI exhibited some typical clinicopathological characteristics. MSI and MMR expression in CRC had good concordance. However, it is still extremely necessary to perform PCR-CE. We recommend that testing packages of different sizes should be developed in clinical practice to create a testing echelon, to facilitate comprehensive selection according to experimental conditions, clinical diagnosis, and treatment needs.

## 1 Introduction

Microsatellite instability (MSI) is often found in colorectal cancer (CRC). MSI is essentially a change in the length of the microsatellite (1). Studies have shown that MSI is mostly caused by mismatch repair (MMR) protein expression deficiency. Thus, MMR protein expression may reflect MSI status. Detection of MMR protein expression by immunohistochemistry (IHC) and DNA-based analysis of MSI status are two different methods for evaluating the same biological responses (2). Polymerase chain reaction-capillary electrophoresis (PCR-CE) is commonly used for direct testing of MSI status, which is also the currently recognized gold standard. MSI has important clinical significance in CRC: it is the preliminary screening step for Lynch syndrome, as well as a biomarker of prognosis and prediction of adjuvant chemotherapy efficacy. Moreover, it is also a predictor of the efficacy of checkpoint inhibitors in advanced solid tumors (3, 4). The ESMO consensus for the management of patients with metastatic colorectal cancer states that MSI testing has strong predictive value for checkpoint inhibitor treatment in metastatic CRC patients (5). The NCCN Clinical Practice Guidelines in Colon Cancer and Rectal Cancer (2019V1) and 2020 CSCO Guidelines for Colorectal Cancer all recommend that MMR protein and/or MSI testing should be performed in all of the CRC patients, so that a suitable treatment regimen and prognosis evaluation are carried out based on the results of these two methods (6). That means developing MSI-related research is important for CRC. Therefore, MSI testing and IHC staining of MMR protein expression were performed on 502 patients with CRC in this study, and the concordance between PCR-CE and IHC results, feasibility, and economic practicality were analyzed to provide experimental data for optimizing detection. The correlation between MSI and various clinicopathological parameters was examined to provide a scientific basis for personalized diagnosis and treatment, as well as personalized treatment efficacy prediction in CRC. This will provide a more comprehensive reference for accurate pathological diagnosis and prognosis evaluation.

## 2 Materials and methods

### 2.1 Materials

A total of 502 patients with a definitive colon or rectal cancer diagnosis in our department between 2019 to 2022 were selected for this study. For a criteria for the inclusion and exclusion of patients enrolled into this study, we excluded patients may also suffer from other diseases that may significantly impact the status of microsatellite instability and mismatch repair protein expression. There were 303 males which accounted for 60.4% and 199 females which accounted for 39.6%. The ages of the patients ranged from 22 years old to 92 years old, and the median age was 63 years old. Clinicopathological parameters such as tumor location, tumor size, gross type, histological classification, differentiation, T-stage, lymph node metastasis and distant metastasis of the patients were also collected.All patients had definitive pathological diagnosis results. Formalin-fixed, paraffin-embedded (FFPE) tissues where tumor tissues accounted for > 50% of the total were selected as the tumor samples. Preparation of 3um slides for immunohistochemical staining. Paracancerous normal FFPE tissues were also selected. The study was conducted in accordance with the Declaration of Helsinki, and approved by the Medical Ethics Committee of Zhejiang Provincial People’s Hospital.

### 2.2 MSI testing and results interpretation

The expert consensus on CRC clinical testing of molecular markers stated that PCR-CE is the gold standard for MSI testing. DNA was extracted and MSI was performed by multiplex fluorescence PCR and capillary electrophoresis gene analysis.In this study, the QIAamp^®^ DNA extraction kit was used to extract DNA from tumor tissues and corresponding healthy colon mucosal tissue. Monitoring DNA concentration and quality with Nanodrop2000 micro nucleic acid assay. The Tongshu MSI testing kit were used to study and an Applied Biosystems 3130 (ABI Inc, USA) were used to detect MSI status. GeneMapperV4.1 software was used to analyze the sequencing results. Based on the recommendations in the Bethesda guidelines, the 2B3D NCI panel was used to test five loci (BAT25, BAT26, D2S123, D5S346, D17S250) to compare the microsatellite status of tumor and normal tissue DNA. High microsatellite instability (MSI-H) is defined as instability in ≥2 loci, low microsatellite instability (MSI-L) is defined as instability in one locus, and microsatellite stable (MSS) is defined as no instability in the five loci. Two experienced molecular pathologists carried out the interpretation.

### 2.3 IHC staining of MMR protein expression and results interpretation

IHC results for MMR protein expression can reflect MMR functional status: proficient mismatch repair (pMMR) is defined as intact MMR protein expression and deficient mismatch repair (dMMR) is defined as deficient MMR protein expression. The slides were baked at 65 degrees for one hour, dewaxed, subjected to antigen repair, and then incubated with appropriate amounts of primary antibody in drops. IHC was done on all tissue samples to detect the loss of MMR protein expression using the following antibodies: MLH1, PMS2, MSH2, and MSH6.All of the primary antibodies used in this study were purchased from the DAKO company; the secondary antibodies were the DAKO EnVisionTM anti-rabbit and anti-mouse universal immunohistochemistry reagents. IHC staining for MLH1,MSH2,PMS2 and MSH6 protein were performed using the primary antibodies at dilutions of 1:200,1:500,1:300 and 1:200 respectively. DAKO AutostainerLink 48 was used for IHC staining. Interpretation criteria: CAP criteria were used to determine if MMR protein expression was deficient: when the internal control (normal intestinal mucosa, tumor interstitial cells, inflammatory cells) nucleus is well-stained, presence of nuclear staining in any confirmed tumor cells is defined as positive expression, and negative expression is defined as no staining in any tumor cell nucleus. pMMR means all four proteins are positive and dMMR is defined as ≥ 1 protein not expressed (7). Two experienced pathologists carried out the interpretation. To achieve consensus, a third experienced pathologist was asked to make a judgment when there was any disagreement.

### 2.4 Statistical analysis

SPSS 22.0 statistical software was used for statistical analysis. Pearson’s Chi-squared test was used for analysis of inter-group differences. Kappa consistency test was used to analyze the concordance between two methods. Sensitivity, specificity, positive predictive value, and negative predictive value were analyzed. For correlation, a difference with a P < 0.05 was considered statistically significant.

## 3 Results

### 3.1 MSI testing results

**Figure 1:**
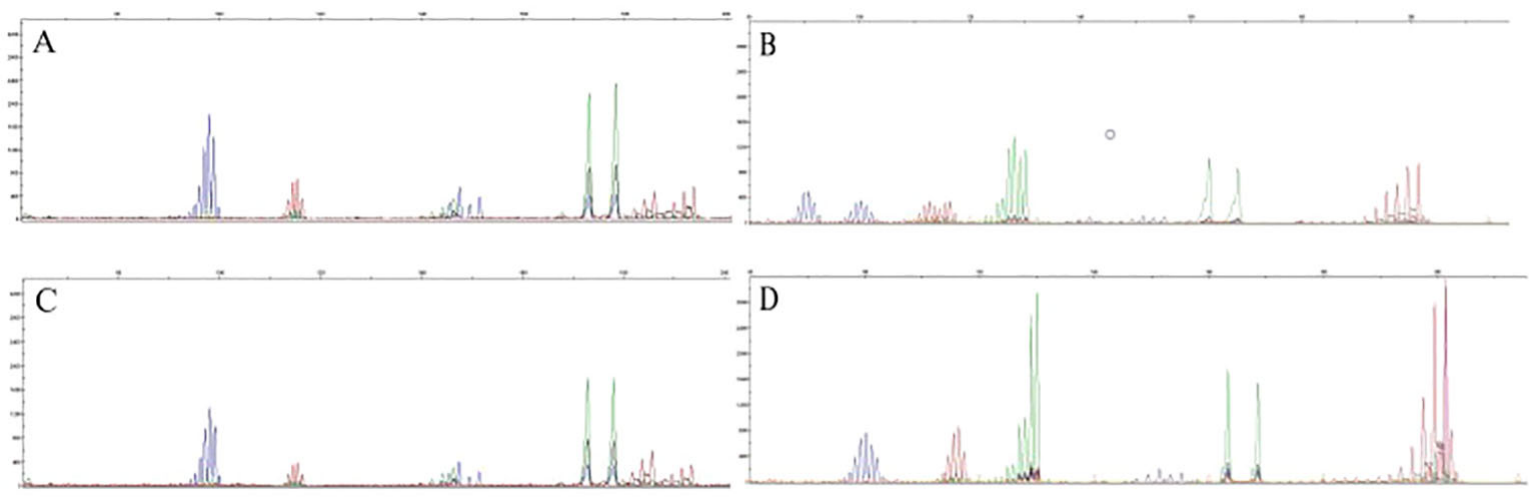
Sequencing maps of MSI by PCR-CE. **(A)** Tumor sample for MSS; **(B)** Tumor sample for MSI-H; **(C)** Paracancerous normal sample for MSS; **(D)** Paracancerous normal sample for MSI-H.

PCR-CE results showed that out of the 502 CRC patients, 64 patients were MSI-H, 19 patients were MSI-L, and 419 patients were MSS. The detection rate for MSI-H was 12.7% and the detection rate for both MSI-L (3.8%)and MSS (83.5%)was 87.3%. In MSI, if the tumor is displaced at several sites compared to normal tissue (cut edge), the site is judged as microsatellite instability. **Figure 1** shows the capillary electrophoresis chromatogram.

### 3.2 MMR protein expression results

**Figure 2:**
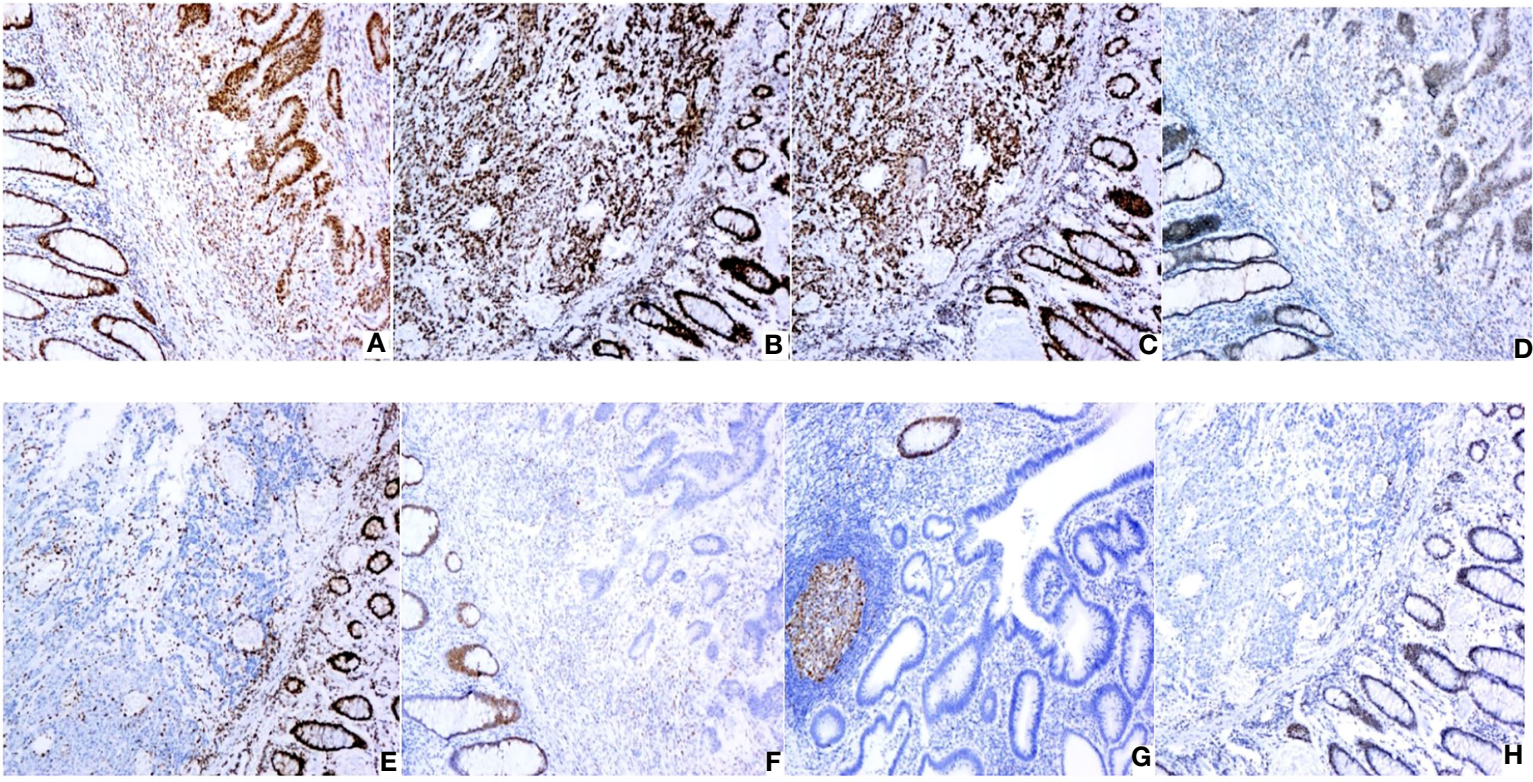
Expression of MMR proteins in CRC (original magnification: × 100): **(A)** Positive MLH1 expression; **(B)** Positive MSH2 expression; **(C)** Positive MSH6 expression; **(D)** Positive PMS2 expression; **(E)** Negative MLH1 expression; **(F)** Negative MSH2 expression; **(G)** Negative MSH6 expression; **(H)** Negative PMS2 expression. IHC staining for MLH1,MSH2,PMS2 and MSH6 protein were performed using the primary antibodies at dilutions of 1:200,1:500,1:300 and 1:200 respectively.

Out of the 502 CRC patients, 430 (85.7%) patients were found to be pMMR and 72 (14.3%) patients were found to be dMMR. In dMMR patients, 43 cases had loss of both MLH1 and PMS2, 11 cases had loss of both MSH2 and MSH6, 7 cases had PMS2 loss alone, 4 cases had MLH1 loss alone, 3 cases had MSH2 loss alone, 2 cases had MSH6 loss alone, 1 case had MLH1 and MSH2 loss, as well as 1 case had MLH1, PMS2, and MSH6 loss. **Figure 2** shows IHC staining, and **Table 1** shows dMMR protein expression deficiency distribution.

**Table 1:** The patterns of dMMR expression in 502 CRC patients.

dMMR Type	n	Percentage
MLH1(-), PMS2(-)	43	59.7%
MSH2(-), MSH6(-)	11	15.3%
PMS2(-)	7	9.6%
MLH1(-)	4	5.6%
MSH2(-)	3	4.2%
MSH6(-)	2	2.8%
MLH1(-), MSH2(-)	1	1.4%
MLH1(-), PMS2(-), MSH6(-)	1	1.4%
Total	72	100%

### 3.3 Analysis of concordance between MSI and MMR expression

According to the guidelines, dMMR in MMR protein expression presents as MSI-H in the PCR-CE testing, where pMMR presents as MSI-L or MSS. The overall concordance of the two methods was 98.4% (494/502) and consistency was good (Kappa = 0.932). There were 8 cases showing dMMR but MSS/MSI-L. The MMR protein expression status of all 8 cases were that only one of the four proteins was defectively expressed while the remaining three were proficiently expressed, including PMS2 (-) in 4 cases, MSH2 (-) in 3 cases, and MSH6 (-) in 1 case. 7 cases were all MSS, while 1 case with defective MSH2 expression was MSI-L. Using PCR-CE as the gold standard testing method, the sensitivity and specificity of IHC were 100% and 98.2%, respectively, and the positive predictive value and negative predictive value were 88.9% and 100%, respectively (**Table 2**).

**Table 2:** Comparison of MSI and MMR expression results in CRC.

MMR	MSI		Total
	MSS/MSI-L	MSI -H	
**pMMR**	430	0	430
**dMMR**	8	64	72
**Total**	438	64	502

### 3.4 Correlation between MSI and clinicopathological parameters

After removing samples with incomplete clinicopathological information, MSI testing results were used as the gold standard for 296 CRC samples with complete parameters. For comparison of clinicopathological parameters, the samples were divided into MSI-H and MSI-L/MSS, according to MSI status. The specific results are shown in **Table 3**. Based on the results, it can be seen that MSI-H was more likely to occur in patients with colorectal cancer compared to women (P = 0.026). The tumor site where MSI-H was more likely to occur was the right hemi-colectum compared to MSS/MSI-L(P < 0.01). Similarly, MSI-H was more likely to occur in patients with colorectal cancer with tumors larger than 5 cm(P = 0.046). Patients with CRC with histology of mucinous adenocarcinoma or adenocarcinoma with mucinous adenocarcinoma had a greater chance of developing MSI-H (P < 0.01). While patients with CRC whose gross type was ulcerated were more likely to develop MSI-H (P = 0.001). Poorly differentiated CRC patients were more likely to develop MSI-H than well differentiated patients(P = 0.022), however, patients with T-stage I/II were more likely to develop MSI-H (P = 0.001), while CRC patients without lymph node metastases and distant metastases were also more likely to develop MSI-H(P < 0.01).No significant correlation between microsatellite instability status and age of onset. Histological classification and grading criteria followed the fifth edition of WHO. The 8th edition of the AJCC recommended staging criteria was used for TMN staging.

**Table 3:** Correlation between clinicopathological characteristics and MSI in CRC.

Parameters	n	MSI		P value
		MSS/MSI-L (%)	MSI -H (%)	
Age (year)				
< 50	31	24 (8.11%)	7 (2.36%)	0.852
≥ 50	265	209 (70.61%)	56 (18.92%)	
Gender				
Male	177	147 (49.67%)	30 (10.13%)	0.026*
Female	119	86 (29.05%)	33 (11.15%)	
Location				
Right colon	68	13 (4.39%)	55 (18.58%)	0.000*
Center colon	102	99 (33.45%)	3 (1.01%)	
Rectum	76	75 (25.34%)	1 (0.34%)	
Others	50	46 (15.54%)	4 (1.35%)	
Tumor size				
< 5 cm	169	140 (47.30%)	29 (9.80%)	0.046*
≥ 5 cm	127	93 (31.41%)	34 (11.49%)	
Histological classification				
Adenocarcinoma	257	222 (75.00%)	35 (11.82%)	0.000*
mucinous adenocarcinoma	25	2 (0.68%)	23 (7.78%)	
Others	14	9 (3.04%)	5 (1.68%)	
Gross type				
Protuberant	117	102 (34.46%)	15 (5.06%)	0.001*
Infiltrating	23	21 (7.09%)	2 (0.68%)	
Ulcerative	156	110 (37.16%)	46 (15.54%)	
Differentiation				
Well	6	3 (1.01%)	3 (1.01%)	0.022*
Moderate	185	154 (52.03%)	31 (10.47%)	
Poor	101	72 (24.32%)	29 (9.80%)	
Undifferentiated	4	4 (1.35%)	0 (0%)	
T stage				
T1–2	62	39 (13.18%)	23 (7.77%)	0.001*
T3–4	234	194 (65.54%)	40 (13.51%)	
Lymph node metastasis				
Without	170	110 (37.16%)	60 (20.27%)	0.000*
With	126	123 (41.55%)	3 (1.01%)	
Distant metastasis				
Without	270	62 (20.95%)	208 (70.26%)	0.000*
With	26	25 (8.45%)	1 (0.34%)	

* It means MSI is significantly correlated with this parameter.

## 4 Discussion

Acquired genome instability is one of the hallmarks of CRC. It mainly includes chromosome instability, MSI, and CpG island methylation (1). In the human genome, microsatellites (MS) are short tandem repeats of 1–6 nucleotides with 10–60 repeats that show high polymorphism due to copy number variation. MSI is defined as changes in MS length due to insertion or deletion of repeats, resulting in new microsatellite alleles. MSI is the main molecular presentation of mismatch repair (MMR) deficiency (2). MMR can maintain genome stability and is one of the main mechanisms of DNA damage repair. Normal MMR function is vital for maintaining genome stability. The MMR gene translation mismatch repair proteins include two families (MutS and MutL). As MMR gene mutation or MLH1 promoter methylation causes MMR deficiency, loss of MMR protein expression due to pathogenic events (including germline mutations, somatic cell mutations, and epigenetic inactivation) causes an inability to repair DNA mismatches, significantly increasing genome instability and increasing spontaneous mutation frequency. This causes aberrant cell proliferation and differentiation, thereby promoting tumorigenesis and tumor progression (3).

The incidence and mortality rate of CRC have been increasing. Recent studies showed that the incidence of MSI in CRC is 10.03%–16%. Bai et al. reported that 10.03% of CRC patients have MSI-H (8). A study published by Ratovomanana et al. found that the incidence of CRC in MSI-H was 16% (9). Younger colorectal cancer patients are less likely to have MSI-H or BRAF mutations than older bowel cancer patients, and BRAF mutation status is highly correlated with MMR protein expression (10). Testing for BRAF, MLH1 promoter methylation and MMR germline genes along with detection of microsatellite instability can distinguish between sporadic CRC or genetic disorders such as Lynch syndrome (11).This study found that in 502 CRC samples, there were 64 (12.7%) patients with MSI-H. Of these, MSI-L and MSS were 19 and 419 cases, respectively, for 87.3% in total, which is consistent with previous studies. The overall concordance of MMR protein expression and MSI was 98.4% (494/502). Although both methods had high consistency (Kappa = 0.932) and are often discussed together in clinical practice, they are not equivalent. dMMR and MSI-H are not simultaneously detected in some patients (12). With regard to MMR protein expression, this study found that 72 (14.3%) patients had dMMR and 430 (85.7%) had pMMR, which was slightly higher than the detection rate for MSI-H. Among them, eight patients had MMR loss when IHC was used and the MSI test results showed MSI-L or MSS. Comparison with the IHC staining results found that MSI-H was not usually detected *via* PCR-CE in some patients with PMS2 or MSH6 loss. This may be because MMR missense mutation causes MMR protein function deficiency but retains its antigenicity. Some CRC patients with deficient MMR function have normal MMR protein expression with IHC testing (13). A previous study reported that dMMR caused by MSH6 mutation has a lower probability of inducing MSI-H and may not meet the criteria for MSI-H diagnosis (14). Further, MSI-H tumors occasionally arise from hitherto undiscovered MMR pathway proteins (15). In addition, MSH6 solitary loss often results in inconsistency between MMR expression and MSI. This is because MSH3 can interact with MSH2 to form MutSβ and partially replace MutSα when MSH6 is lost, thus carrying out the function of recognition and resulting in the correction of some DNA mismatch errors. Thereby, the result does not present as MSI-H (16). This may also be due to functional overlap between MMR proteins (PMS2 and PMS1, MSH6, and MSH3). Therefore, although protein expression is lost, the IHC result shows dMMR and PCR shows MSS (17). PMS2 solitary loss will result in false positive IHC staining results in some patients, as its matching MLH1 seeks out other MMR proteins to form dimers to carry out its effects (18).

The high concordance between MSI and MMR protein expression needs to be established on a reliable and validated testing platform (particularly for IHC interpretation). IHC is easy to operate, inexpensive, easy to set up, does not require tumor cell content, has its own internal control, and can be used as a preliminary screening method. However, quality control for IHC techniques must be strengthened from sample collection, fixation, and staining. For example, primary antibody retrieval conditions and concentrations, as well as DAB color development duration control, must be ideal to avoid poor tissue fixation, insufficient or excessive antigen retrieval, or non-specific staining caused by different antibody clone numbers, inaccurate cell localization, and deviations in staining intensity. IHC preprocessing must comply with relevant regulations and interpretation standards must be unified. The CAP criteria are recommended. Different pathologists will carry out double-blind review to avoid interpretation errors caused by individual subjective differences (19). PCR-CE can compensate for the limitations of IHC. In this study, the five loci recommended by the Bethesda panel were tested. A previous report also recommended testing six or up to 10 mono- and dinucleotide loci to increase sensitivity and specificity (20). As the gold standard, PCR-CE is used to test MSI status, but it cannot determine MMR gene changes.

Aside from these two methods, gradual advances in and accumulation of more data and validation in next generation sequencing (NGS) will allow it to become an effective method for microsatellite testing. It may be used to supplement the gold standard and increase diagnostic accuracy. A study combined CRC chromosome morphological characteristics and AI deep learning to effectively identify chromosome characteristics using an artificial intelligence algorithm, thereby determining the microsatellite status of patients. Non-invasive blood ctDNA and fecal sample tests have also been proposed. Continuous exploration of new test methods will help promote understanding of CRC occurrence and progression mechanisms.

This study found that compared with MSI-I/MSS, MSI-H was found more in CRC patients who were female (P = 0.026) and had right hemi-colon disease (P < 0.01), a maximum tumor diameter ≥ 5 cm (P = 0.046), ulcerative disease (P = 0.001), mucinous adenocarcinoma or adenocarcinoma with mucinous component (P < 0.01), poor differentiation (P = 0.022), T stage I/II (P = 0.001), and no lymph node and distal metastasis (P < 0.01), which is consistent with previous studies. However, there have been no other reports on gross tumor type or tumor size. One of the studies noted that age of onset of CRC with microsatellite instability is low, however, our study did not validate this correlation with age. This may be due to ethnic and regional differences between studies.

## 5 Conclusions

In summary, this study proposed that the PCR-CE testing for MSI is extremely necessary and should be generally promoted in clinical practice. Even though MSI and MMR protein expression showed good concordance in CRC, MSI testing still cannot be completely replaced by MMR protein expression assessment. In addition, it is recommended that the pathology departments should develop testing packages of different sizes based on clinical needs to form a testing echelon. All CRC patients should first undergo MMR protein expression testing with IHC staining. After that, MSI testing should be performed in suspected Lynch syndrome patients by PCR-CE. These two tests can be performed simultaneously when the patient can afford them. Furthermore, NGS may be performed thereafter for validation when efficacy prediction will be carried out before immunotherapy, and prognosis evaluation will be carried out before personalized treatment. We recommend that a echelon of testing packages should be established so that selection can be based on experimental conditions, clinical diagnosis, and treatment needs.

## Data availability statement

The datasets presented in this study can be found in online repositories. The names of the repository/repositories and accession number(s) can be found below: yemeihua@hmc.edu.cn.

## Ethics statement

The studies involving human participants were reviewed and approved by Medical Ethics Committee of Zhejiang Provincial People’s Hospital. Written informed consent for participation was not required for this study in accordance with the national legislation and the institutional requirements.

## Author contributions

Conceptualization: MY and XH. Data curation: GR and HY. Formal analysis: XH and SL. Investigation: GR. Methodology: MY and LQ. Resources: MY. Validation: HY and LQ. Writing – original draft: MY. Writing – review and editing: MY and SL. All authors contributed to the article and approved the submitted version.
